# Impact of Prior Periacetabular Osteotomy on Total Hip Arthroplasty Outcomes in Patients with Developmental Dysplasia of the Hip

**DOI:** 10.3390/jcm14113661

**Published:** 2025-05-23

**Authors:** Adam Czwojdziński, Jakub Leśniak, Andrzej Sionek, Dariusz Grzelecki, Jarosław Czubak

**Affiliations:** 1 Department of Orthopedics, Pediatric Orthopedics and Traumatology, Centre of Postgraduate Medical Education, Professor Adam Gruca Orthopedic and Trauma Teaching Hospital, Konarskiego 13, 05-400 Otwock, Poland; 2 Department of Orthopedics and Rheumoorthopedics, Centre of Postgraduate Medical Education, Professor Adam Gruca Orthopedic and Trauma Teaching Hospital, Konarskiego 13, 05-400 Otwock, Poland

**Keywords:** hip, arthroplasty, dysplasia, DDH, THA, PROM, mHHS

## Abstract

**Background:** This study aims to demonstrate the challenges during the treatment of patients with osteoarthritis due to the development of dysplasia, which can be faced by surgeons who treat patients with THA. Our objective is to present our findings from a comparison of patients who underwent total hip arthroplasty (THA) for osteoarthritis secondary to developmental dysplasia of the hip (DDH), with or without a prior periacetabular osteotomy (PAO). **Methods:** We divided patients into two groups. Group A was adolescents who underwent periacetabular osteotomy and further THA in the orthopedic center (29 hips), and Group B was adolescents who underwent THA without PAO but suffered from DDH (24 hips). We measured blood loss, cup size, cup positioning, cup coverage, inclination and anteversion of the cup, duration of surgery, clinical scores, time of hospitalization, and the presence of ossification. **Results:** The mHHS score demonstrated significant differences (group A: 89 points vs. group B: 91 points; *p* = 0.03). The blood loss was expressed in the difference in Hb concentration preoperatively and on the 3rd postoperative day, which was higher by 0.85 mg/dL in group A (group A: 4.4 mg/dl vs. group B: 3.55 mg/dL; *p* = 0.004). Also, the value of HCT on the 3rd postoperative day was significantly lower in group A (group A: 27% vs. group B: 29.5%; *p* = 0.02). Radiological measurements showed a statistically significant difference in the Brooker scale (*p* = 0.005). Thirteen patients from group A after THA expressed first-grade ossifications or higher, and two patients from group B had first- and second-grade ossifications. Furthermore, a notably larger distalization was observed after the PAO procedure compared to those who underwent the procedure without PAO previously (79 mm [IQR = 73–83 mm] vs. 74.5 mm [IQR = 69–77 mm]; *p* = 0.004). Conclusions: Patients reported lower mHHS results, a higher risk of heterotopic ossifications, prosthesis head distalization, and more significant blood loss during the perioperative period after THA in patients who underwent PAO compared to those without a previously performed osteotomy.

## 1. Introduction

Development dysplasia of the hip (DDH) is a congenital disease that can occur with a broad spectrum of radiological and clinical symptoms. Despite properly treating dysplasia early, its residual form may develop in up to one-third of patients [1]. In patients who meet radiological criteria, different osteotomies may be performed to increase the survival of the native hip joint. One common procedure is periacetabular osteotomy (PAO) using the Bernese method, described by Professor Ganz in 1988 [2,3]. This procedure relies on reorienting the acetabulum of the hip joint to restore proper joint anatomy, which leads to a change in the action of forces, improves biomechanics, and consequently reduces pain and increases the survival of the joint itself. Good results of PAO in patients who ultimately met the radiological indications were widely described [4,5,6,7], which encouraged the performance of this procedure in a broad group of patients with residual DDH at different ages [4]. Comparable outcomes were observed at the authors’ institution, which has over 20 years of experience in performing PAO (Ganz osteotomy). Witnessing the positive outcomes of PAO, different authors proposed to extend the indications in young adults and perform osteotomy, even in those who did not entirely meet the criteria, to save a native joint and postpone THA. Nonetheless, in patients with osteoarthritis (OA) secondary to developmental dysplasia of the hip (DDH), total hip arthroplasty (THA) is considered the treatment of choice in cases of advanced joint destruction, even at a relatively young age, due to its consistently positive clinical outcomes [8,9,10,11]. It has an impact on the trend of extending the indications to PAO. However, there is a specific group of patients who present indications and contraindications to performing both methods; this group is young patients with DDH and moderate clinical symptoms who fulfill the radiological criteria but present a moderate grade of OA that justifies qualification to THA (borderline to perform PAO) [12]. In this group of patients, the orthopedic surgeon has to choose which method should be performed according to the procedure survival, long-term outcomes, and potential complications. Due to frequent qualification doubts in borderline patients with DDH, it is justified to demonstrate the consequences of previously performed PAO, as it can either complicate or facilitate subsequent total hip arthroplasty (THA), potentially affecting radiological, functional, and clinical outcomes.

This study aims to compare clinical and radiological outcomes in patients after THA is performed due to OA secondary to residual DDH (and not treated surgically before) with those who previously underwent PAO, presented OA progression, and qualified for the THA. A key question guiding this study is whether a previously performed periacetabular osteotomy (PAO) adversely affects the clinical and radiological outcomes of subsequent total hip arthroplasty (THA). To ensure the reliability of the results, this study focused on a homogeneous cohort consisting exclusively of patients who underwent total hip arthroplasty (THA) at a comparable age and had pre-existing indications for both periacetabular osteotomy (PAO) and THA prior to the initial surgical intervention, representing the borderline group.

## 2. Materials and Methods

This retrospective study included patients (from 18 to 60 years old) operated on in a single orthopedic center from 2013 to 2022. The decision on the method of surgical treatment, which treatment was performed first, PAO or THA, was made together with the patient and was mainly based on the patient’s expectations (about preserving his/her own joint) and the intensity of pain. All patients that underwent THA due to osteoarthritis that occurred secondary to residual DDH (I grade Crowe classification) [13], with or without previously performed PAO. Patients who underwent PAO were deemed suitable for THA due to an unsuccessful outcome following osteotomy, progression of osteoarthritis, and pain in the joint. The exclusion criteria were previously performed surgical procedures on the hip joints other than PAO, people with cerebral palsy, autoimmune chronic inflammatory diseases (e.g., rheumatoid arthritis), those with a history of active cancer, and those who had suffered a fracture of the hip joint. This is a retrospective study, and all patients lost to follow-up were excluded from the analysis.

**Figure 1:**
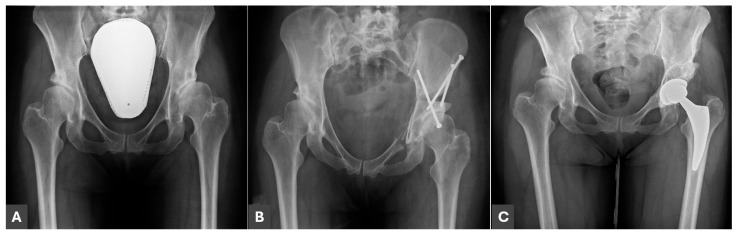
A 45-year-old female patient with residual DDH. (**A**) AP radiograph before PAO; (**B**) AP radiograph after PAO of the left hip; (**C**) AP radiograph after screw removal and THA subsequently to PAO.

**Figure 2:**
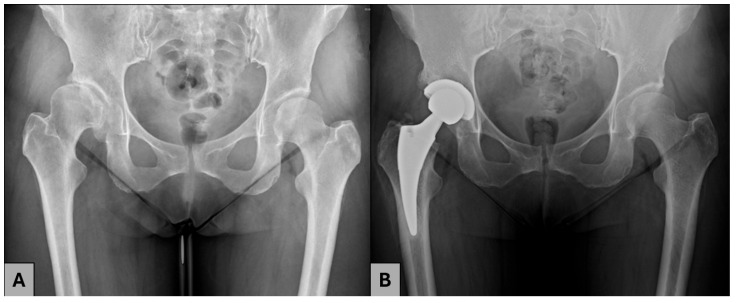
A 44-year-old female patient with residual DDH. (**A**) AO radiograph before THA; (**B**) AP radiograph after THA.

Finally, 49 patients (53 hip joints) qualified for the study and were divided into two groups. Group A included 25 patients (29 operated hip joints) who previously underwent PAO and subsequent THA (Figure 1). Group B (control group) contained 24 patients (24 hip joints) with DDH and subsequent osteoarthritis who underwent THA without any previous surgical treatment (Figure 2).

**Figure 3:**
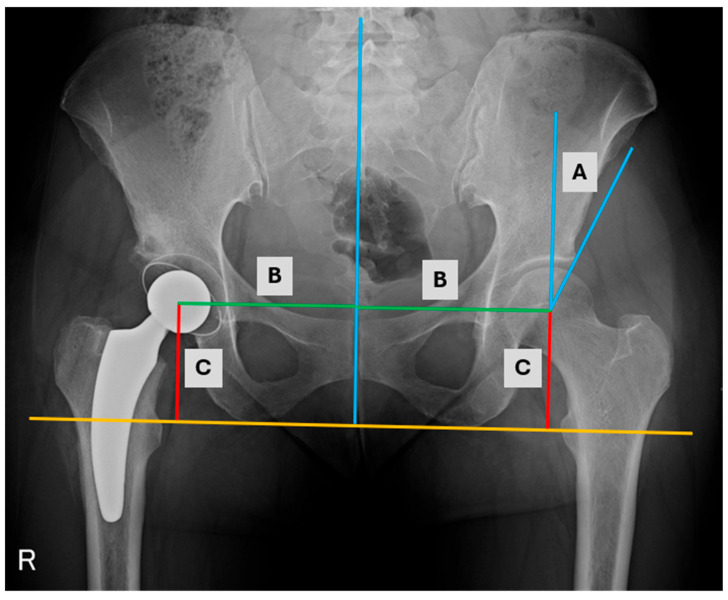
AP radiograph of the hips with measurements (A) Wiberg angle, (B) medialization, (C) distalization.

Preoperative demographical factors such as gender, age, weight, body mass index (BMI), and clinical characteristics such as baseline hemoglobin (Hb) values, hematocrit (HTC), modified Harris hip score (HHS), Oxford hip score (OHS) and types of implants were analyzed (Table 1 and Table 2). Preoperative radiological evaluation was based on AP radiographs. Radiological measurements, such as femoral head coverage (FHC), medialization, distalization, and Wiberg angle, were calculated for all patients (Figure 3, Table 3). Postoperative radiological outcomes were assessed on the AP radiographs on the first day after the surgery and during ambulatory visits. Patients had a minimum 1-year follow-up, including one follow-up visit at least 12 months after THA. The same clinical and radiological outcomes in the first postoperative day were determined in all patients. THAs via posterolateral approach with the reconstruction of the rotator cuff muscles and the joint capsule was performed in all patients with no drain after surgery. Cementless endoprostheses were used, and the aim of the cup implantation was to achieve an inclination of 35–50 degrees and 10–15 degrees anteversion. (Table 1) In all patients, there was no need to use screws. Standard antibiotic prophylaxis (2.0 g IV cefazoline) and antithrombotic prophylaxis with low molecular weight heparin were administered according to recommendations.

Hospitalization time (from the day of surgery to the day of discharge), duration of surgery, blood loss determined by the drop in Hb concentration, medialization, distalization, cup implant size of acetabular endoprosthesis part, bone coverage of the acetabulum (bone coverage index (BCI)), angle of inclination, acetabular anteversion according to Lewinnek, heterotopic ossification assessment according to Brooker scale [14], mHHS, and OHS were compared between two the examined groups.

Statistical analysis was performed using Excel 2019 (Microsoft) and Statistica 13.3 (Tibco). Demographic and clinical continuous variables were presented as the median and the interquartile range (IQR). The Shapiro–Wilk test was used to determine the normality of the data distribution. Because of the lack of normality of the data and relatively small groups, statistical differences were assessed using the Mann-Whitney U and Fisher exact test (2 × 2, 3 × 2). The level of significance was set at *p* < 0.05.

The study had institutional review board approval from The Centre of Postgraduate Medical Education, 18 November 2015, (83/PB/2015) and was performed according to Helsinki’s 1967 Declaration of Law.

## 3. Results

The groups were homogeneous in terms of preoperative demographic and clinical factors, and no significant differences were found. The difference in the FHC results is attributable to the periacetabular osteotomy, which was performed with the primary aim of improving this parameter, among others. The postoperative mHHS score (measured at the last follow-up visit, between 35 and 65 months after surgery) demonstrated significant differences (group A: 89 points vs. group B: 91 points; *p* = 0.03). The blood loss expressed in the difference in Hb concentration preoperatively and on the third postoperative day, which was higher by 0.85 mg/dL in group A (group A: 4.4 mg/dl vs. group B: 3.55 mg/dL; *p* = 0.004); also the value of HCT on the third postoperative day was significantly lower in group A (group A: 27% vs. group B: 29.5%; *p* = 0.02). (Table 4).

Radiological measurements showed a statistically significant difference in the Brooker scale (*p* = 0.005). Thirteen patients from group A after THA expressed first-grade ossifications or higher, and two patients from group B had first- and second-grade ossifications. Moreover, significantly greater distalization was observed in patients who had undergone a prior PAO compared to those without a previous PAO (79 mm [IQR = 73–83 mm] vs. 74.5 mm [IQR = 69–77 mm]; *p* = 0.004) (Table 5).

Postoperative complications such as prosthesis dislocation, periprosthetic joint infections, fractures, neurological deficits, and thromboembolic events were not observed in both analyzed groups.

## 4. Discussion

Although THA is the gold standard in treating degenerative hip joint disease, considering joint-saving procedures, especially in young patients, is justified. PAO is one of the options to prevent or delay osteoarthritis progression and may increase the patient’s quality of life, especially in adolescents. Due to encouraging postoperative outcomes, indications for PAO in middle-aged people and those with higher-grade osteoarthritis secondary to DDH are being extended. However, it may negatively impact and accelerate the joint degeneration process and patients may require early conversion to THA. In this study, we exposed that patients who require conversion to THA after PAO achieved significantly lower scores in mHHS and expressed more and higher-grade heterotopic ossifications compared to patients who underwent THA without PAO. Moreover, higher blood loss expressed in Hgb and HCT drops were measured between preoperative results and the third postoperative day. Notably, the PAO group required more transfused blood units, with twelve units compared to just two units in the THA group.

The appearance of heterotopic ossifications may be an essential problem in the group of patients after THA, and their frequency varies from 5 to 87% [15,16]. Ossifications requiring treatment due to limitation of range of motion, pain, and swelling (grade III and IV on the Brooker scale) are less frequent, and their incidence is up to 12% [16]. Several risk factors predisposing to heterotopic ossifications were identified in the literature [17]. Only a few research studies have correlated previously performed hip surgery, such as trochanteric osteotomy or fracture treatment around the hip joint [15,16]. Although previously performed PAO was not emphasized as a risk factor, our research found statistically significant more frequent incidents compared to the group of patients who underwent THA without a prior osteotomy. However, only grade I and grade II in Brooker scale ossifications were observed, which usually do not require surgical excision and additional radiation or drug medication treatment. Moreover, prolonged duration of the surgery, intraoperative blood loss, and hematoma formation are also potential risk factors for heterotopic ossification [18,19]. We have observed significantly higher blood loss and longer but statistically insignificant time of surgery when THA was performed after PAO, which may indirectly correlate with the expression of heterotopic ossification formation. Despite the prolonged operation time, no correlation with clinical outcome was observed.

Radiological, clinical, and functional assessment after pelvic osteotomy was investigated in several studies [20,21]. Kołodziejczyk et al. revealed that Ganz PAO improved radiological and clinical results whether or not the hip surgery was performed in childhood [22]. De La Rocha et al. observed the same trend in radiological and functional results after PAO when pelvic osteotomy was performed before those without previously performed osteotomy [20]. However, several risk factors of PAO failure were described in the meta-analysis conducted by Sambandam et al., who indicated a significant impact of change in postoperative lateral center edge (*p* < 0.05) [5]. They also revealed that the odds of THA increase by 22% when subluxation occurs, 9% when additional proximal femoral osteotomy (PFO) is needed, and 54% with late PFO. Novais et al., in a retrospective study, revealed that patients > 40 years old with Tönnis grade 2 and poor preoperative dysfunction have a high risk of PAO failure [6]. Other studies emphasized the influence of different factors such as narrowing of the joint space width [23], presence of an os acetabuli and severe grade osteoarthritis [24], and increased BMI [25]. We agree that, in young patients with DDH and a painful joint who ultimately meet the qualification criteria and do not respond to the non-operative treatment, PAO is the best solution that improves clinical and functional outcomes and hip survival [4,7,26].

Regarding patients with borderline indications for PAO, particularly young adults with residual DDH and moderate hip osteoarthritis, it is essential to consider which treatment option—osteotomy or THA—should be chosen on an individual basis. In the patients qualified for PAO according to extended indications, the risk of failure increases, and after conversion to THA, the results may be worse. Osawa et al. have observed significantly poorer outcomes in HHS, ROM, and cup placement of THA after failed PAO [27]. This result aligns with our findings, where mHHS was significantly lower in patients who underwent THA after PAO than in those who had not had an osteotomy before. Although several studies revealed lower outcomes in clinical and functional scales after THA with previously performed different pelvic osteotomies [27,28,29], other studies did not confirm these trends [30,31,32]. Therefore, there is a need to conduct further prospective studies with randomization on larger cohorts to determine whether previously performed pelvic osteotomies have a negative impact on subsequent THA.

We found a statistical difference in distalization, which partially aligns with other studies that demonstrate differences, particularly in cup positioning after osteotomy. Komiyama et al. tended to insert the acetabular component more proximal and lateral and observed larger bone coverage and acetabular sizes in patients who previously underwent PAO [33]. Similarly, Ma et al. observed that, after PAO, an increased cup size may be implanted, improving its stability [33]. Correct positioning of the cup implant in dysplastic hip joints is challenging, especially in those with higher grade osteoarthritis, higher grade DDH in Crowe classification, and in patients who previously underwent PAOs [21,34,35]. We believe recreating proper hip biomechanics directly associated with prosthesis position is crucial to achieving prolonged implant survival, good clinical and functional outcomes, and patient satisfaction.

This study has some limitations that should be considered before analyzing the results. First is the retrospective character, and the small size of the examined groups is evident. However, it is related to the restrictive inclusion criteria used to achieve the most reliable results and answer the question of whether PAO negatively impacts the outcomes of subsequently performed THA. The second limitation is that the minimum 2-year follow-up is short (median 51.5 and 53 months in both groups). We know there is a need to conduct longer follow-ups to assess long-term results, especially in THA after the PAO group. The third limitation is that, although we measured blood loss by checking the drops of Hb and Htc, we did not measure blood loss during surgery, which comes with the inconvenience of weighing gauze pads. Due to this study’s retrospective nature, we cannot reproduce this data. Lastly, postoperative radiological outcomes were analyzed according to AP radiograms of both hips. Measurements taken from CT scans would be more accurate and may lead to a precise assessment of the 3D position of the prosthesis components, including cup anteversion. Cup implantation is crucial in the context of osteoarthritis stemming from DDH, particularly due to the observed lack of the anterior wall of the acetabulum. Comparing THA with and without prior PAO may yield differences between the two groups. This study measured anteversion using a less precise Lewinnek method based on the AP radiograms. CT scans are not routinely performed due to higher radiation doses, which is not advisable, especially in younger patients.

In the future, studies could be expanded to include randomized controlled trials to strengthen the evidence and continue the study to assess the wear of implants and long-term complications in both groups

## 5. Conclusions

Total hip arthroplasty (THA) following periacetabular osteotomy (PAO) is associated with greater blood loss, longer operative time, higher risk of ossification, and a lower mHHS clinical scale score compared with primary THA. These factors should be carefully considered, especially by surgeons planning surgery, and discussed, particularly in patients with borderline indications for PAO who may have a higher risk of failure and subsequent conversion to THA after osteotomy.

## Figures and Tables

**Table 1:** Types of implants.

Implant Type	Group A (n = 29) (THA After PAO)	Group B (n = 24) (THA Without PAO)
Stem (cementless)		
CLS	12	3
Fitmore	14	11
Optymis	3	7
Accolade	0	2
Modulus	0	1
Cup (cementless)		
Allofit	26	14
RM	3	7
Trident	0	2
Delta	0	1

**Table 2:** Preoperative demographical and clinical factors. Continuous variables were presented as medians with IQR.

	Group A (n = 29) (THA After PAO)	Group B (n = 24) (THA Without PAO)	*p*-Value
Male hips/Female hips	0/29	0/24	-
Age during THA (years)	41.2 (36.1–45.9)	44.8 (43–49)	0.17 *
Body mass (kg)	63 (58–70)	70 (63–78)	0.076 *
BMI (kg/m^2^)	23.8 (22–27.1)	24.9 (23.1–30.4)	0.27 **
Baseline concentration values of Hb (g/dL)	13.7 (13–14.3)	13.65 (12.9–14.2)	0.96 *
HCT (%)	41 (39–43)	41 (39–43)	0.4 *
Follow-up (months)	53 (35–63)	51.5 (31–59)	0.63 *
Modified Harris Hip Score	55.4 (52.7–56)	56 (55.4–56.6)	0.064 *
Oxford Hip Score	19 (18–19)	19 (18–20)	0.095 *

* Mann-Whitney U test, ** Student *t*-test.

**Table 3:** Preoperative radiological measurements.

Variable	Group A (n = 29) (THA After PAO)	Group B (n = 24) (THA Without PAO)	*p*-Value
FHC (%)	71 (65.4–80)	60.95 (55.4–64)	<0.001 *
Medialization before THA (mm)	111 (102–120)	113 (104–119)	0.9 *
Distalization before THA (mm)	82 (75–87)	79.5 (72–83)	0.17 *
Wiberg angle (°)	12.8 (8.8–18)	15 (10–18)	0.4 *

* Mann-Whitney U test.

**Table 4:** Postoperative clinical results.

Variable	Group A (n = 29) (THA After PAO)	Group B (n = 24) (THA Without PAO)	*p*-Value
Time of hospitalization (days)	4.4 (3.7–4.8)	3.6 (2.9–4.3)	0.20 *
Hgb drop (preoperative vs. 3rd postoperative day) (g/dL)	4.4 (3.6–4.8)	3.6 (2.9–4.3)	0.004 *
HCT 3 days after the surgery (%)	27 (25–30)	29.5 (27–31)	0.02 *
Time of surgery (min)	75 (60–85)	65 (58.8–70)	0.20 *
Modified Harris Hip Score	89 (83–91)	91 (91–91)	0.03 *
Oxford Hip Score	48 (46–48)	48 (48–48)	0.19 *
Duration between PAO and THA (months)	72 (37–87)	—	—

* Mann-Whitney U test.

**Table 5:** Results of postoperative radiological measurements.

Variable	Group A (n = 29) (THA After PAO)	Group B (n = 24) (THA Without PAO)	*p*-Value
Brooker scale (0/1/2/3/4)	16/10/3/0/0	22/1/1/0/0	0.005 *
Medialization THA (mm)	91 (88–95)	92.5 (87–99)	0.53 **
Distalization THA (mm)	79 (73–83)	74.5 (69–77)	0.004 **
Inclination (°)	48.1 (41–52.5)	46.8 (43–51.6)	0.88 **
Anteversion (°)	22.3 (15.7–28.7)	21.6 (16.7–33.6)	0.53 **
Bone Coverage Index (%)	84.8 (78.7–100)	92.05 (81.4–100)	0.27 **
Cup implant size (mm)	48 (45.5–50)	48 (47.5–48)	0.83 **

* Fisher exact test (2 × 3); ** Mann-Whitney U test.

## Data Availability

All data generated or used during this study are available from the corresponding author and first author upon reasonable request.

## Abbreviations

THA	Total Hip Arthroplasty
PAO	Periacetabular
DDH	Developmental Dysplasia of the Hip
OA	Osteoarthritis
MHHS	Modified Harris Hip Score
OHS	Osteoarthritis
BCI	Bone Coverage Index
BMI	Body Mass Index
HGB	Hemoglobin
HCT	Hematocrit
IQR	Interquartile range
PFO	proximal femoral osteotomy
ROM	Range of motion
FHC	Femoral Head Coverage
